# Enhanced anti-tumor efficacy with multi-transgene armed mesenchymal stem cells for treating peritoneal carcinomatosis

**DOI:** 10.1186/s12967-024-05278-5

**Published:** 2024-05-15

**Authors:** Yoon Khei Ho, Jun Yung Woo, Kin Man Loke, Lih-Wen Deng, Heng-Phon Too

**Affiliations:** 1 Department of Biochemistry, National University of Singapore, Singapore, 117596 Singapore; 2 NUS Centre for Cancer Research (N2CR), Yong Loo Lin School of Medicine, National University of Singapore, Singapore, Singapore; 3 AGeM Bio, Singapore, 119276 Singapore; 4 Singapore Innovate, Singapore, 059911 Singapore

**Keywords:** Mesenchymal stem cells, Non-viral gene modification, Peritoneal carcinomatosis

## Abstract

**Background:**

Mesenchymal stem cells (MSCs) have garnered significant interest for their tumor-tropic property, making them potential therapeutic delivery vehicles for cancer treatment. We have previously shown the significant anti-tumour activity in mice preclinical models and companion animals with naturally occurring cancers using non-virally engineered MSCs with a therapeutic transgene encoding cytosine deaminase and uracil phosphoribosyl transferase (CDUPRT) and green fluorescent protein (GFP). Clinical studies have shown improved response rate with combinatorial treatment of 5-fluorouracil and Interferon-beta (IFNb) in peritoneal carcinomatosis (PC). However, high systemic toxicities have limited the clinical use of such a regime.

**Methods:**

In this study, we evaluated the feasibility of intraperitoneal administration of non-virally engineered MSCs to co-deliver CDUPRT/5-Flucytosine prodrug system and IFNb to potentially enhance the cGAS-STING signalling axis. Here, MSCs were engineered to express CDUPRT or CDUPRT-IFNb. Expression of CDUPRT and IFNb was confirmed by flow cytometry and ELISA, respectively. The anti-cancer efficacy of the engineered MSCs was evaluated in both in vitro and in vivo model. ES2, HT-29 and Colo-205 were cocultured with engineered MSCs at various ratio. The cell viability with or without 5-flucytosine was measured with MTS assay. To further compare the anti-cancer efficacy of the engineered MSCs, peritoneal carcinomatosis mouse model was established by intraperitoneal injection of luciferase expressing ES2 stable cells. The tumour burden was measured through bioluminescence tracking.

**Results:**

Firstly, there was no changes in phenotypes of MSCs despite high expression of the transgene encoding CDUPRT and IFNb (CDUPRT-IFNb). Transwell migration assays and *in-vivo* tracking suggested the co-expression of multiple transgenes did not impact migratory capability of the MSCs. The superiority of CDUPRT-IFNb over CDUPRT expressing MSCs was demonstrated in ES2, HT-29 and Colo-205 *in-vitro*. Similar observations were observed in an intraperitoneal ES2 ovarian cancer xenograft model. The growth of tumor mass was inhibited by ~ 90% and 46% in the mice treated with MSCs expressing CDUPRT-IFNb or CDUPRT, respectively.

**Conclusions:**

Taken together, these results established the effectiveness of MSCs co-expressing CDUPRT and IFNb in controlling and targeting PC growth. This study lay the foundation for the development of clinical trial using multigene-armed MSCs for PC.

**Supplementary Information:**

The online version contains supplementary material available at 10.1186/s12967-024-05278-5.

## Background

Peritoneal carcinomatosis (PC) is represented by advanced metastases of digestive-tract and gynaecological cancer cells to the peritoneal lining, resulting in malignancy in the peritoneal cavity. It is considered a terminal disease due to high recurrence and poor prognosis with overall survival (OS) of 3–6 months. Patients may receive vigorous combinatorial treatment consisting of cytoreductive surgery (CRS) and hyperthermic intraperitoneal chemotherapy (HIPEC), which has been reported to improve OS to 15–70 months [1–3]. Nonetheless, even after treatment, a significant number of patients develop recurrence, resulting in an overall 5 year survival ranging from 11 to 19% in PC patients [4–7]. Unfortunately, CRS and HIPEC involve major procedures with considerable mortality and morbidity [8]. Benefits of the iterative procedures may be limited with patients already compromised by late-stage cancer [5, 9]. Despite the lack of survival benefits, systemic chemotherapy is the standard treatment for recurrences after CRS and HIPEC [4, 10]. Intraperitoneal regional treatments to achieve continuous high local concentrations of multimodal cytotoxic agents is a critical factor to improve clinical outcome for patients with recurring PC.

The rationale of intraperitoneal administration is to maximize the chemotherapeutic dosage delivered directly into the peritoneal tumour nodules while minimizing systemic toxicity [11, 12]. For the same intent, stem cells have been explored as cellular vehicles for delivery of therapeutic agents in targeting peritoneal cancers [13–16]. Although yet to be fully elucidated, MSCs have been widely accepted as a promising vehicle due to their inert immunogenicity and natural tumour-trophic properties [17–19]. Furthermore, engineered MSCs could potentially serve as biofactories to provide continuous therapeutics to the tumour milieus. We and others have reported prolonged and sustained expression of therapeutic genes in engineered stem cells for more than 7 days [20, 21]. Proof-of-concept and acceptable levels of safety have already been reported in multiple phase I trials for peritoneal cancer treatment where MSCs were engineered to deliver various therapeutic agents including enzyme for prodrug conversion [22], interferon beta (IFNb) [23], and oncolytic virus (NCT02068794).

Prodrug systems offer a safer option compared to traditional chemotherapy because the non-toxic prodrugs are converted into active drugs locally, avoiding systemic toxicities [24, 25]. We developed a highly efficient cationic polymer-based transfection method to engineer MSCs to express a therapeutic transgene—cytosine deaminase uracil phosphoribosyl-transferase fused to a green fluorescent protein (GFP) reporter. These engineered cells showed strong anti-cancer potency in *in-vitro,* in subcutaneous laboratory models [20, 26] and in companion animals with naturally occurring cancers [27]. The non-toxic prodrug (5-flucytosine, 5FC) is converted by CD into 5-flurouracil (5FU) that disrupts the nucleotide biosynthesis, leading to apoptosis [28, 29]. Interestingly, emerging evidence suggests the full therapeutic potential of 5FU is through the involvement of the cGAS-STING pathway. This signalling cascade reaction elicits the production of type I IFNs from cancer cells, leading to the activation of innate immunity and long-lasting anti-tumour effects [30, 31]. Lee et al. has shown that the cGAS-STING pathway plays a critical role in converting immunologically “cold” peritoneal tumours to immunologically favourable phenotypes in a type I IFN-dependent manner, resulting in the eradication of tumour and ascites [32].

Recent findings showed that STING signalling is habitually defective in ovarian cancer [33, 34], colorectal cancer [35, 36] and gastric cancer [37] and is often associated with poor prognosis. Exogeneous type I IFN treatment may potentially be a way to overcome STING deficiency and enhance the therapeutic efficacy of 5FU. Apart from the critical role in modulating immune responses [38], Type I IFN is also known to have a direct effect on cancer cells [39] and can potentiate 5FU cytotoxicity by inducing S-phase accumulation and apoptosis [40–43]. In clinical studies, recombinant IFNs in combination with 5FU have been shown to be effective in some [44–47], but not in others [48, 49]. Significant challenges limiting the clinical benefits include the issues of systemic toxicities and short half-life of recombinant IFNb [41, 50]. Thus, systemic treatment will unlikely attain therapeutically meaningful local concentrations necessary to synergise with 5FU effects. An interesting approach is the use of MSCs as an effective targeting vehicle to deliver a 5FU prodrug and IFNb to tumor sites, thereby increasing the local therapeutic concentrations [51, 52]. In the present study, we showed that non-viral engineered MSCs co-expressing CD prodrug system and IFNb is a promising approach to treat PC.

## Methods

### Cell culture

Human adipose tissue-derived MSCs from a single donor (#100225) were purchased from Essent Biologics (Centennial, CO). The MSCs were cultured in complete culture media consisting of α-MEM supplemented with human platelet lysate (Mill Creek Life Sciences, USA). To maintain experimental consistency, only MSCs between passages 3–5 were used in this study. Cancer cell lines A549 (human lung adenocarcinoma; ATCC CCL-185) was maintained in DMEM supplemented with 10% FBS (HyClone), HT-29 (human colorectal adenocarcinoma; kindly provided by Johnny Ong Chin-Ann, National Cancer Centre Singapore) in McCoy’s 5A medium supplemented with 10% FBS, whereas Colo-205-LUC-GFP (human colon carcinoma; GeneCopoeia SCL-C05-HLG) and ES2-LUC-tdT (human ovarian clear cell carcinoma; kindly provided by Deng Lih Wen, National University of Singapore) were maintained in RPMI 1640 media supplemented with 10% FBS. All cell cultures were maintained in a humidified incubator at 37 °C in 5% CO_2_.

### Construction of the plasmid

A multi-cistronic vector encoding CDUPRT-IFNb was cloned into an existing expression vector bearing CDUPRT as previously described [20]. All plasmids were purified using the E.Z.N.A Plasmid DNA Maxi Kit (Omega Bio-Tek).

### Transfection and expression analysis

Transfection was carried out in 6-well plate format. Plasmid complex, at a total volume of 20 μL/cm^2^, consist of 250 ng/cm^2^ DNA, 1.1 µL/cm^2^ Polyethylenimine MAX (Polyscience; 1 mg/mL) and DPBS, was incubated at room temperature for 15 min. The plasmid complex was then added dropwise into MSCs (150000 cells/cm^2^ in 6-well plates) supplemented with 500 ng/mL Lipofectamine^™^ 2000 Transfection Reagent (ThermoFisher Scientific) and 0.5 μM Vorinostat (Histone deacetylase inhibitor; HDACi, BioVision) in complete medium. The culture media was replaced with fresh media at 24 h post-transfection. Then, cells were further incubated for 24 h before analysis. Cell images were taken with EVOS FL Cell Imaging System (Thermo Fisher Scientific) equipped with a GFP (Ex470/Em510) fluorescent light cube. For flow cytometric analyses, cells were washed by DPBS, trypsinized using TrypLE Express. Percentage of fluorescent positive cells was quantified by Attune NxT Flow Cytometer system (ThermoFisher Scientific), and the raw data was analysed using non-modified MSCs as the negative control at < 0.8%, using Invitrogen Attune NxT software (Version 3.1.2, ThermoFisher Scientific).

### Trilineage differentiation

A trilineage differentiation assay was used to evaluate the trilineage differentiation potential of MSCs to give rise to chondrocytes, osteoblasts and adipocytes using a commercially available differentiation media (StemPro^™^ Differentiation Kits, Thermo Fisher Scientific). For chondrogenic differential assay, cells were plated at a density of 10^6^ cells/well in ultra-low attachment 96-wellplate and then induced using the chondrogenic differentiation media after 24 h. The differentiation media were replaced twice weekly. After 23 days, differentiation was assessed by Alcian Blue solution staining of sulfated proteoglycans. For osteogenic and adipogenic differential assays, cells were plated at a density of 100,000 cells/well in 24well-plates and replaced with corresponding differentiation media the next day. After 16 days, adipogenic differentiation was assessed by Oil Red O solution staining of oil droplets. For osteogenic differentiation, differentiation was assessed by Alizarin red S solution staining of calcium deposits at day 23 post induction. Images were taken at 4 × objective for chondrogenesis and 20 × objective for both osteogenesis and adipogenesis using a Nikon Eclipse Ts2-FL microscope (Nikon Instruments Inc., NY, USA).

### Immunophenotyping

Flow cytometry analysis of the cells was performed using 4% formaldehyde fixed cells and stained according to the manufacturer’s protocol. Briefly, the cells were washed with DPBS and detached with TrypLE Express. Then the cells were fixed in 4% formaldehyde for 15 min at room temperature, washed twice with DPBS and resuspended at a concentration of 1 × 10^6^ cells in 100 µL staining buffer. The cells were stained with antibodies to CD73 (APC, Invitrogen, clone AD2), CD90 (APC, Miltenyi Biotec, clone REA897), CD105 (APC, Invitrogen, clone SN6), CD14 (PE, Miltenyi Biotec, clone REA599), CD19 (PE, Miltenyi Biotech, clone REA675), CD34 (APC, Invitrogen, clone 4H11), CD45 (APC, Invitrogen, clone HI30), HLA-DR (PE, Miltenyi Biotec, clone REA805) and corresponding isotypic controls (PE and APC, Miltenyi Biotec, clone REA293; APC, Invitrogen, clone P3.6.2.8.1). The cells were incubated in the dark for 15 min, washed, resuspended in DPBS, and analysed immediately. The immunophenotyping analysis was performed using a CytoFLEX LX flow cytometer (Beckman Coulter). At least 10,000 events were acquired in each sample and analyzed using the Attune ^™^ NxT Software (Version 3.1.2, Thermo Fisher Scientific).

### *In-vitro* studies

#### Coculture experiment

500 cells/well for Colo-205-LUC-GFP, 2000 cells/well for HT29 and ES2-LUC-tdT were seeded in 96-well plates. Four hours later, CDUPRT_MSCs or CDUPRT-IFNb_MSCs were plated to the cancer cell culture at a ratio of 1 MSC to 1, 6, 12, 25, 50, 100, and 200 cancer cells. After overnight attachment of the cells, the medium was replaced with fresh cancer cell medium supplemented with or without 150 μg/mL 5FC (TCI Chemicals). Five days later, cell viability was measured by MTS assay with at least five biological replicates for each condition.

#### Mitochondrial membrane potential and apoptosis assay

CDUPRT_MSC or CDUPRT-IFNb_MSC were co-cultured with ES2 at a ratio of 1 MSC to 1 cancer cell. Two days after the addition of 150 μg/mL 5FC, all cells were harvested and stained with TMRE (biotium) or annexin-V (PE Annexin V Apoptosis Detection Kit with 7-AAD, Tonbo Biosciences) according to the manufacturer’s instructions. Percentage of fluorescent positive cells was quantified by Attune NxT Flow Cytometer system (ThermoFisher Scientific), and the raw data was analysed using Invitrogen Attune NxT software (Version 3.1.2, ThermoFisher Scientific).

#### Migration assay

Migration assay was performed using 24-transwell plates (Corning) with 8.0 µm pore size inserts (Corning). A total of 200,000 cancer cells were seeded in the lower chamber with complete medium. Four hours later, the complete medium was discarded and washed twice with DPBS and replaced with serum free medium. A total of 100,000 MSCs were seeded in the upper chamber with serum free medium. After 48 h, MSCs were fixed in 4% formaldehyde. Unmigrated MSCs were removed using a Q-tip and migrated MSCs that penetrated the porous membrane were stained with Hoechst 33342 (5 µg/mL), documented with a fluorescence microscope (EVOS M7000 Imaging System, Thermo Fisher Scientific) and quantified using EVOS Analysis software (Version 1.5.1479.304, Thermo Fisher Scientific).

### *In-vivo* studies

Five to six-week-old female athymic nude (CrTac:NCr-Foxn1^nu^) mice (InVivos) were purchased and all animal experiments were carried out in accordance with relevant guidelines and regulations. General anesthesia in mice was performed by isoflurane inhalation. The research protocol was reviewed and approved by the National University of Singapore, Institutional Animal Care and Use Committee (IACUC; protocol number R18-1383).

### In-vivo tumour tropism

To develop *in-vivo* model of tumour tropism, 1 × 10^6^ ES2-LUC-tdT cells suspended in 100 µL of Plasma-Lyte were injected intraperitoneally into the lower right quadrant of the abdomen. All mice including the control mice without ES2-LUC-tdT were treated with MSCs overexpressing *Renilla* luciferase injected into the peritoneal cavity and monitored for 21 days. Tumour tropism of MSCs was monitored using an in vivo imaging system (IVIS^®^ Spectrum In Vivo Imaging System, PerkinElmer) after substrate injection (ViviRen^™^, Promega; 1 mg/kg per mouse). The images were analyzed using the Living Image software (PerkinElmer). All mice were euthanized at experimental endpoint.

### In vivo cytotoxic effect of CDUPRT_MSC/5FC and CDUPRT-IFNβ_MSC/5FC

To develop in vivo model of peritoneal carcinomatosis, a total of 1 × 10^6^ ES2-LUC-tdT cells were suspended in 100 µL of Plasma-Lyte and carefully injected into the peritoneal cavity using an insulin syringe. Three days after tumour cell implantation, mice were divided into three groups, unmodified MSC, CDUPRT_MSC and CDUPRT-IFNb_MSC. For treatment, 1 × 10^6^ MSCs suspended in 100 μL of Plasma-Lyte were injected intraperitoneally into the abdomen. One day post-MSC administration, mice were treated intraperitoneally with 500 mg/kg/day of 5FC for four consecutive days. A total of two treatment cycles were administered. Mice were anaesthetized by isoflurane inhalation, and tumor growth was monitored using an in vivo imaging system (IVIS^®^ Spectrum In Vivo Imaging System, PerkinElmer) after luciferin injection (D-Luciferin, PerkinElmer; 50 mg/kg per mouse). The photon flux of each mouse was measured using Living Image software (PerkinElmer). Organs were isolated after the mice were euthanized at the end of the experiment.

### Statistical analysis

All experiments were repeated at least thrice. Parametric Student *t*-test was used for statistical analysis using Excel (Microsoft). For the *in-vitro* coculture studies (Fig. 4), the qPCR study (Additional file: 8) and the THP1 assay (Additional file: 9), the data was analyzed using Graphpad Prism. Statistical significance was categorized as p < 0.05 and data were reported as mean ± SD.

## Results

### Sustainable and high expression of dual therapeutic proteins by non-virally engineered allogeneic MSCs

**Fig. 1:**
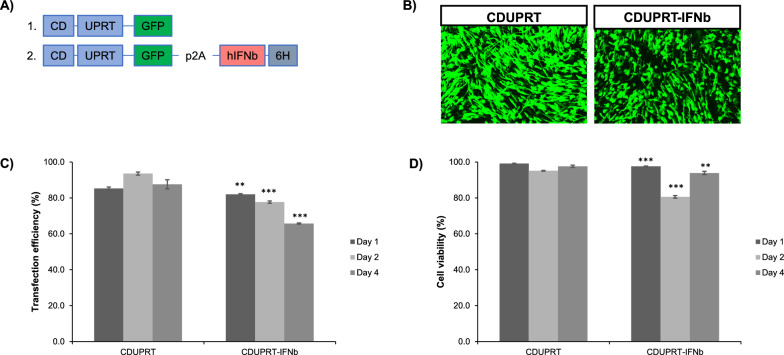
Modification of MSC to overexpress CDUPRT and IFNb. **A** A multi-cistronic vector encoding CDUPRT-IFNb (construct 2) was cloned into an existing expression vector bearing CDUPRT. **B** Representative images of MSCs transfected with CDUPRT and CDUPRT-IFNb on day 1 post transfection. **C** Transfection efficiency and (**D**) cell viability of cells on day 1, 2, and 4 post-transfection. All bar charts and line graphs are represented as mean ± SD of biological triplicates (n = 3). Statistical significance was measured using a two-tailed Students’ t-test to compare the CDUPRT and CDUPRT-IFNb groups. A p-value of less than 0.05 was considered significant. (*p < 0.05, **p < 0.01, ***p < 0.001)

Next, We assessed the transfection efficiency and quality of adipose tissue-derived MSCs after transfected with plasmids encoding either the therapeutic transgene, CDUPRT or CDUPRT- IFNb. Specifically, a “self-cleaving” 2A peptide was used for the coexpression of IFNb downstream of CDUPRT [53]. The CDUPRT transgene (also referred to as the “prodrug system”) is tagged with GFP, serving as a reporter (Fig. 1A). In compliance with the Food and Drug Administration (FDA) guidelines (21 CFR 1271.85) the MSCs were screened and cleared of the various pathogens and cultured in xenofree media to avoid the risks of exposure to animal derived pathogens [54].

Optimization of transfection parameters (DNA and polymer amount) for both the constructs were guided by design of experiments (DOE). Leveraging on the non-viral transfection method developed in our previous study [55], an optimal condition of the modification of MSCs was identified (Additional file 1). At DNA/polymer ratio of 250 ng/1.1 µL per cm^2^ for both CDUPRT and CDUPRT-IFNb plasmids, more than 80% of the population expressed the transgenes on day 1 after transfection, and this did not adversely affect cell viability. It is worthy to note that cell morphology was unaffected by the high transgene expression (Fig. 1B). Cells and conditioned media were collected to determine protein expression and secretion of CDUPRT and IFNb by western blot and ELISA, respectively. Western blot analysis confirmed expression of CDUPRT in the cytosol, and IFNb was secreted into the culture medium (Additional file 2). IFNb concentration in the culture medium was 494.5 ± 10.4 ng/mL for the CDUPRT-IFNb group, which was significantly higher than that in the CDUPRT or native group (p < 0.01) (Additional file: 3). The conditioned media collected from the cells transfected with CDUPRT-IFNb was subjected to an established functional assay [56]. Upregulation of interferon-stimulated genes (ISGs) in a A549 cell line model confirmed the activity of IFNb secreted by the engineered MSCs (Additional file: 4).

As we intend for modified MSCs to serve as biofactories for sustainable delivery of therapeutic agents at the peritoneal cancer site, prolonged intracellular transgene expression is essential. Hence, we examined the duration of expression for the intended therapeutic agents. Notably, the high intracellular CDUPRT expression, as indicated by GFP^+^ expression was retained over a period of 4 days post transfection (Fig. 1C).

### Engineered MSCs retain genetic stability and immunomodulatory properties

**Fig. 2:**
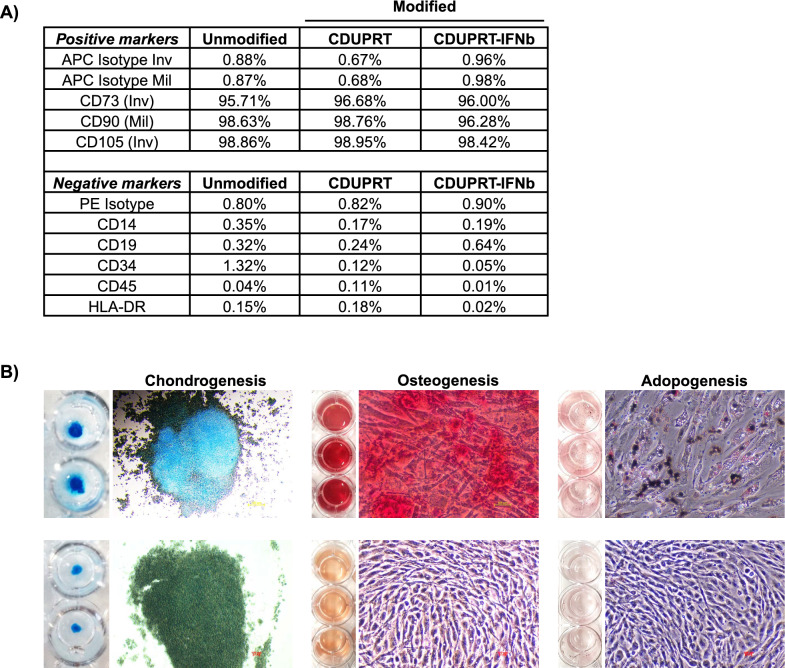
Highly modified CDUPRT-IFNb_MSC retain MSC phenotypic markers and trilineage differentiation potential. **A** Modified and unmodified MSCs were collected and analysed using immunophenotyping for the expression of positive (CD73, CD90 and CD105) and negative (CD14, CD19, CD34, CD45 and HLD-DR) makers. Respective isotype controls were used as the negative gates for flow cytometry analysis. **B** CDUPRT-IFNb modified cells were also subjected to trilineage differentiation. Chondrogenic, osteogenic, and adipogenic differentiation was carried out using respective differentiation medium (top panel). Images on the bottom panel represent the undifferentiated controls after staining with respective solutions. Adipogenic differentiation was assessed after 16 days using Oil Red O to stain for oil droplets. Chondrogenic and osteogenic differentiation were assessed after 23 days using Alcian Blue to stain for sulphated proteoglycans present in chondrocytes, and Alizarin Red S to stain for calcium deposits, respectively

Genetic stability is an essential quality control measure for cell therapy [57]. To ensure the genetic stability of the engineered MSCs, the karyotypes of the engineered MSCs expressing CDUPRT or CDUPRT-IFNb were analysed and compared against unmodified, native MSCs. All three MSC types displayed a normal human karyotype with no detectable cytogenetic changes (Additional file: 5). With unmodified MSCs as reference, the modified MSCs were assessed according to the criteria as stipulated by the International Society for Cell and Gene Therapy (ISCT) [58]. MSC identity was validated as > 95% population expressing CD73, CD90, CD105, and < 2% of the cells expressing CD14, CD19, CD34 and CD45 (Fig. 2A). Notably, the engineered MSCs showed a negligible expression of Human Leukocyte Antigen-DR (HLA-DR), confirming that the MSCs retain low immunogenicity despite the high expression of CDUPRT or IFNb (Fig. 2A). It was also gratifying to note that MSCs transfected with CDUPRT-IFNb retained their chondrogenic, osteogenic, and adipogenic differentiation potential (Fig. 2B), like our previous observations with MSC engineered to express CDUPRT [20].

### *Engineered MSCs migrate and effectively target PC cells *in vitro* and *in vivo

**Fig. 3:**
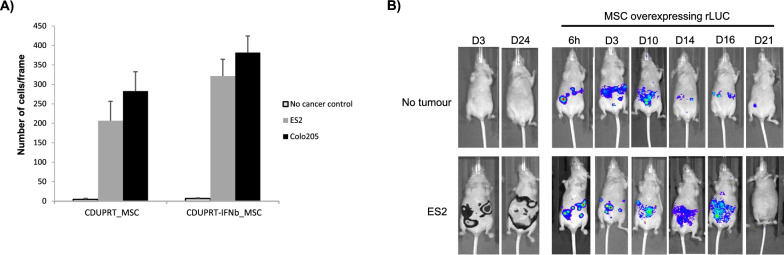
Modified MSCs exhibit efficient tumour tropism in-vitro and in-vivo. **A** CDUPRT_MSC or CDUPRT-IFNb_MSC were subjected to Boyden chamber assays using ES2 or Colo-205. At the end of the assay, the cells were fixed in 4% paraformaldehyde. The unmigrated cells were removed using a Q-tip and migrated cells on the flipside of the insert were stained with Hoechst 33342 and quantified manually. A total of 3 images per insert were obtained from 3 replicate wells per condition. The fold-difference was calculated using a negative control with no cancer cells within the bottom chamber. The data is presented as mean fold-difference ± SEM from triplicate (n = 3) wells. **B** *In-vivo* tumour tropism was evaluated using a nude mouse model with 1 × 10^6^ ES2 cancer cells injected into the peritoneal cavity. Once the tumours were established, MSCs overexpressing Renilla luciferase (rLUC) were injected into the peritoneal cavity and monitored at the indicated time periods

The migratory ability of MSCs toward tumours makes them a highly attractive vehicle for the delivery of therapeutic proteins to target cancers. For the treatment to be successful, the migratory potential of highly modified MSCs need to be intact. To that end, we assessed the tumour tropism of modified MSCs *in-vitro* and *in-vivo*. Relative to a no-cancer control, we found that MSCs modified with CDUPRT or CDUPRT-IFNb were able to selectively migrate towards Colo-205 and ES2 cancer cells (Fig. 3A). An *in-vivo* model of peritoneal carcinomatosis was established via peritoneal injection of ES2 stably expressing firefly luciferase. MSCs modified to express Renilla luciferase were injected into the peritoneal cavity and monitored for 21 days. Interestingly, we observed that the modified MSCs colocalised with the tumours as early as 6 h post-injection and remained in the mice for > 2 weeks. As time progressed, the Renilla luciferase signal spread along the abdomen, indicative that the MSCs were able to track and spread along with the tumours as they progressed. By day 21, the majority of the modified MSCs had cleared from the mice (Fig. 3B). In line with other studies [59, 60], MSCs found to be associated with tumours were detectable for significantly longer durations compared to non-tumour bearing mice.

### *MSCs expressing CDUPRT-IFNb is superior in targeting PC cell lines *in vitro

**Fig. 4:**
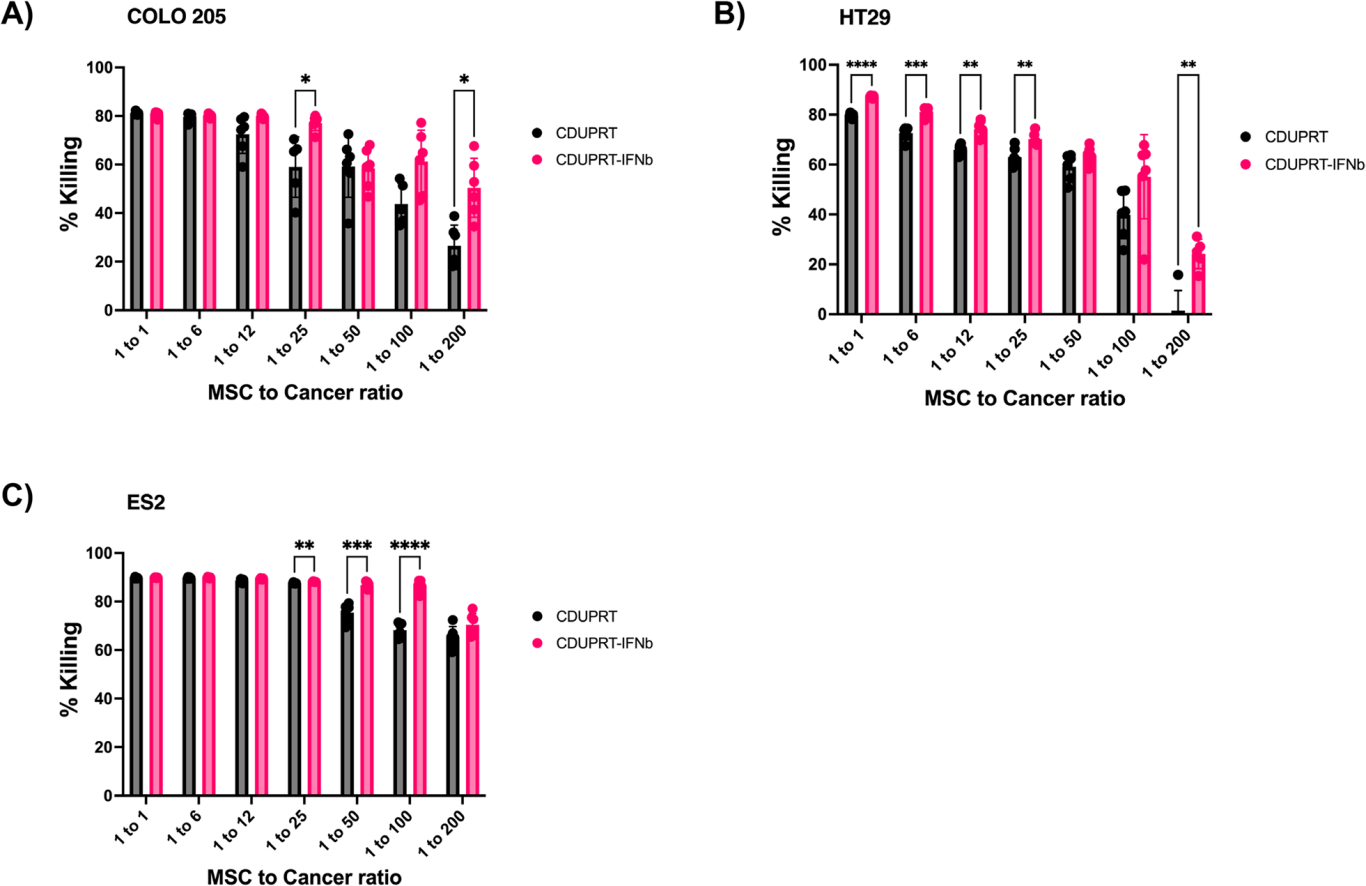
CDUPRT-IFNb_MSCs are highly effective against multiple cancer cell lines. **A** Colo-205, **B** HT-29, and **C** ES2 were co-cultured with CDUPRT-IFNb_MSC or CDUPRT_MSC at 1 MSC to 1, 6, 12, 25, 50, 100, and 200 cancer cells. After one day in co-culture, cultures were supplemented with 150 μg/mL 5FC. Five days later, cell viability was quantified by standard MTS assay. Cells co-cultured with CDUPRT_MSC and CDUPRT-IFNb_MSC at different ratios without 5FC treatment were used as the respective controls to calculate cancer killing efficacy using the formula: % Killing = 1-(sample/control) * 100%. All graphs were represented as mean ± SD from at least five biological replicates (n = 5). Statistical significance was measured using an unpaired Students’ t-test. A p-value of less than 0.05 was considered significant. (*p < 0.05, **p < 0.01, ***p < 0.001, ****p < 0.0001)

Next, we tested the therapeutic potential of both engineered MSCs on various PC cell lines. When we co-cultured Colo-205, HT-29, and ES2 cell lines with MSCs expressing CDUPRT or CDUPRT-IFNb in the presence of the prodrug 5FC, we observed a significant reduction in cell viability (Fig. 4). With 2% of engineered MSCs, a ~ 70% reduction in cell number was observed in all 3 PC cell lines. Remarkably, MSCs expressing CDUPRT-IFNb exhibited enhanced potency compared to CDUPRT when 0.5% engineered MSCs were present in the coculture. Notably, Colo-205 colorectal carcinoma cells co-cultured with MSCs modified with CDUPRT-IFNb at a ratio of 1 MSCs to 200 cancer cells (0.5% engineered MSCs) showed a killing efficiency of approximately 60%, double the percentage of killing of cells modified with CDUPRT alone (p < 0.05, Fig. 4A).

We next demonstrated the in vitro conversion of 5FC to 5FU by the engineered MSCs. High-performance liquid chromatography/electrospray ionization/tandem mass spectrometry analysis of the culture media 1 day after addition of 5FC in the MSC cultures suggested a conversion of 5FC to 5FU at approximately 0.02 µM/engineered MSC/day (Additional file 6). A concentration increase of 5FU in the supernatant was detected with increasing number of engineered MSCs in the presence of 100 µg/mL of 5FC. At 1 CDUPRT_MSC to 50 cancer cells, 1 µM of 5FU/day generated in this condition resulted in ~ 70% cell reduction (Fig. 4). To gain insight into the MSC treatment efficacy relative to 5FU alone, we further determined the dose response of 5FU in ES2 cell line. Here, 75% reduction of ES2 was measured in the presence of 12.5 µM of 5FU (Additional File: 7). The stronger potency with engineered MSCs is likely due to the close proximity release of 5FU to the cancer population, supporting the notion of local drug delivery for effective cancer treatment [61, 62].

**Fig. 5:**
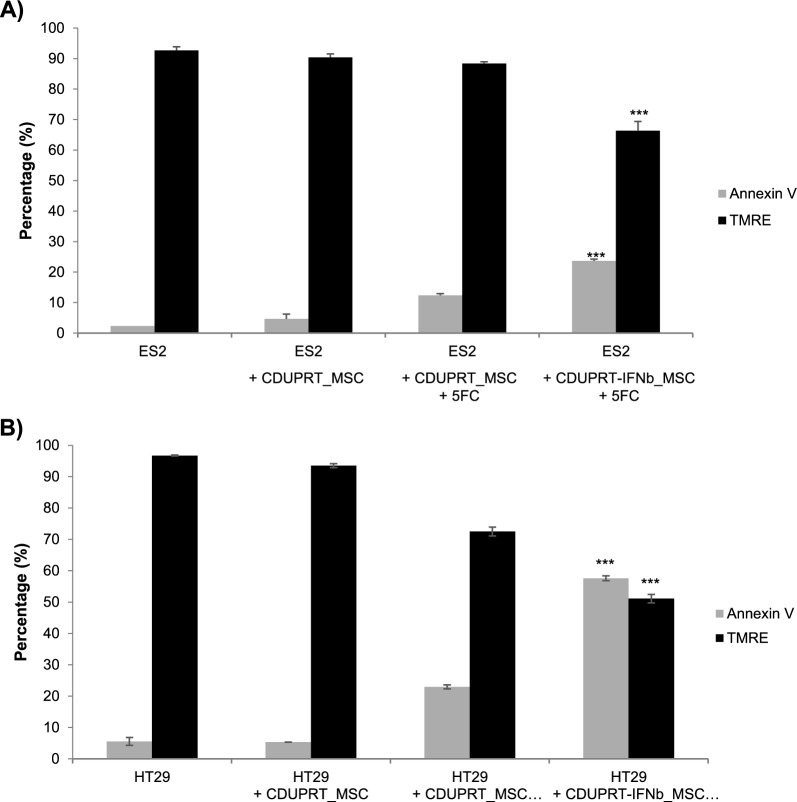
CDUPRT-IFNb_MSC with 5FC disrupts mitochondrial potential and increases apoptosis. CDUPRT_MSC or CDUPRT-IFNb_MSC were co-cultured with (**A**) ES2 or (**B**) HT29 at a ratio of 1 MSC to 1 cancer cells. Two days after the addition of 5FC, all cells were harvested and stained with TMRE or annexin V. The graph represents the percentage of cells positive for TMRE and annexin V presented as mean ± SD from at least three biological replicates (n = 3). Unstained controls were used as the negative gate. Statistical significance was measured using a two-tailed Students’ t-test relative to the CDURPT_MSC + 5FC condition. A p-value of less than 0.05 was considered significant. (***p < 0.001)

To further investigate the potential anti-cancer mechanisms with the CDUPRT-IFNb modified MSCs, we stained the cells on the second day of the coculture study with tetramethylrhodamine ethyl ester perchlorate (TMRE) and annexin-V to examine the changes in mitochondrial membrane potential and apoptosis, respectively. ES2 and HT29 cells undergo significantly greater mitochondrial perturbation in the presence of IFNb, potentially contributing to the increased apoptosis compared to other conditions (Fig. 5). This is consistent with another report that similarly showed mitochondrial perturbation in other ovarian cell lines when cocultured with MSC overexpressing IFNb [16]. IFN has been shown to trigger cell death via TRAIL [63], which acts as an effector of mitochondrial perturbation [64, 65]. To corroborate these findings, qPCR analysis indicated an increment in the expression of TRAIL, DR5 and ISGs (Additional file: 8) in ES2 exposed to the conditioned media of CDUPRT-IFNb_MSC + 5FC treatment, which was significantly higher than that observed with CDUPRT_MSC + 5FC treatment. While the current study focuses on exploring the use of MSCs to deliver 5FU and IFNb, it is interesting to note that a preliminary study using the monocytic cell line THP1 showed that IFNb overexpression led to an enhancement in immune cell polarization (Additional file: 9). This aspect is currently being pursued in greater detail.

### In vivo* evaluation of anticancer efficiency of the engineered MSCs*

Extending the study, we next injected MSCs expressing CDUPRT or CDURPT-IFNb intraperitoneally into a laboratory mice model for peritoneal carcinomatosis. A similar administration route for engineered MSCs with IFNb is currently under phase I clinical trial for treatment of patients with ovarian cancer [23]. Female nude mice (CrTac:NCr-Foxn1^nu^) bearing peritoneal human ES2-tdTomato/Luc tumours were divided into 3 groups, the first group served as a control using native MSCs, while the other 2 groups were treated with CDUPRT or CDUPRT-IFNb expressing MSCs to compare the co-delivery of 5FU and IFNb. One day after the cells were injected peritoneally, 5FC was administered daily for 4 consecutive days to enable the conversion to 5FU. The treatment cycle of MSCs and 5FC was repeated on day 7 and the study was terminated at Day 14 due to the accumulation of excessive ascites in the control group.

**Fig. 6:**
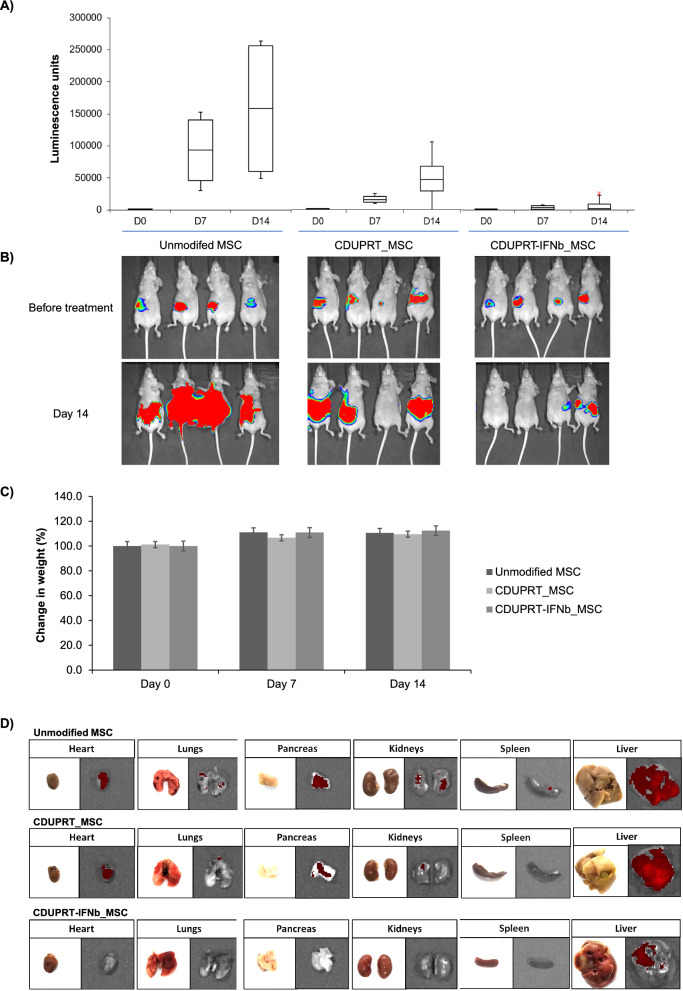
CDUPRT-IFN_MSC exerts a strong tumour suppressive effect in-vivo. The in-vivo model of peritoneal carcinomatosis was established using ES2 by injecting 1 × 10^6^ cancer cells into the peritoneal cavity of nude mice. Tumour burden was assessed using bioluminescence and determined to be approximately similar prior to the start of the experiment. A total of two treatment cycles were administered, each consisting of 1 × 10^6^ MSCs followed by 4 consecutive days of 500 mg/kg/day 5FC via intraperitoneal injection. The treatment cycles were spaced a week apart. Day 0 indicates the day of injection of MSCs for the first treatment dose. The mice were divided into three groups of four mice (n = 4) each. The mice were treated with unmodified MSC, CDUPRT_MSC or CDUPRT-IFNb_MSC. Tumour burden was assessed on day 7 and day 14. **A** The bioluminescence readings from each group presented as a box-and-whisker plot. **B** Bioluminescence images showing tumour burden in mice before treatment and at the end of the study (day 14). **C** Change in weight of the mice throughout the course of the study. **D** After the mice were euthanised, internal organs were harvested and examined for metastatic lesions

To ensure a non-biased assessment of the therapeutic efficacy, the mice were grouped to ensure comparable average tumour burden prior to the start to treatment. The tumour burden was tracked by weekly measurement of bioluminescence reading. Significant suppression of tumour growth was observed in the treatment groups but not in the control group (Fig. 6). Remarkably, animals treated with CDUPRT-IFNb modified MSCs experienced minimal tumour progression compared to the control group treated with unmodified MSCs. Mice treated with cells modified with CDUPRT but not CDUPRT-IFNb experienced a slight increase in tumour burden (Fig. 6A, B). All mice were observed every 2 days post-treatment and scored for any debilitating signs of pain and stress, including laboured breathing, obvious illness, hunched posture, and the ability to remain upright. None of these adverse signs were detected throughout the experiment. However, two mice in the control group were shown to have developed ascites, a commonly observed symptom of end-stage PC, at day 14. Additionally, none of the mice experienced significant loss of weight throughout the experiment (Fig. 6C). At experimental endpoint, the internal organs of the mice were extracted and imaged individually for any signs of luciferase expressing ES2 tumours. Metastases were detected in the heart, lungs, pancreas, kidneys, spleen, and liver in the control group but was less apparent in the treatment groups. Further analysis of the internal organs confirmed the greater therapeutic potential of CDUPRT-IFNb expressing MSCs over its counterparts in suppressing cancer metastasis (Fig. 6D). In this group, metastasis was only found in liver and not in other organs. In the liver, the tumour burden was significantly lower than the other two groups. Potentially, the therapeutic efficacy could be enhanced by increasing the dose of engineered MSCs or number of treatment cycles.

## Discussion

In the recent years, there has been growing interest in utilizing MSCs as cell vehicles to deliver therapeutic agents for localized solid tumour treatments, including patients with advanced peritoneal cancers [66]. Particularly, two phase I clinical trials (NCT02530047, NCT02068794) have successfully demonstrated safety and promising clinical benefits with engineered MSCs administered through intraperitoneal infusion in the cohorts of recurrent ovarian cancer patients [17, 19]. Leveraging on our non-viral method for MSCs modification, we explored the feasibility of co-delivering a 5FU prodrug system and IFNb for localized cytoreductive effect against peritoneal cancers. Our results demonstrated the highly efficacious therapeutic benefits of the combination of 5FU and IFNb. This paves the path for further development towards investigational new drug studies for the application of a phase I clinical trial. This further requires the use of MSCs that are free of various pathogens and are cultured in xenofree media.

Historically, 5FU has been used as the first-line chemotherapeutic for patients with PC of gastrointestinal and colorectal origin [67, 68], and experimentally for advanced ovarian cancer patients [69–71]. To improve therapeutic outcome, 5FU is delivered intraperitoneally to achieve minimal toxicity, higher drug concentration and prolonged half-life of the drug [67, 71]. Nonetheless, some groups have reported that patients receiving intraperitoneal or intravenous administration developed similar adverse effects [72, 73] and the half-life of intraperitoneal 5FU remains short (40 min) [74]. Additionally, the high drug concentration in the peritoneal fluid may not equate to increment in drug penetration into the tumour nodules [75]. Such a lack of therapeutically meaningful local concentrations of systemically administered 5FU and IFNb has contributed to the failure of randomized human trials [41, 50].

Using MSCs to deliver therapeutic agents may circumvent some of these issues as MSCs are known to home and nest onto the tumour site to continually release therapeutic payloads locally [17, 19, 76]. We showed that the homing capacity of the highly overexpressed CDUPRT and CDUPRT-IFNb modified MSCs were comparable, a contrast to a previous report of the reduced migratory property of MSCs post modification [77]. While other delivery systems rely on paracellular or transcellular transport [78], MSCs present unique properties in the ability to penetrate deeply into the central region of tumour mass [79, 80]. Notably, we found that the MSCs were present along with the tumour as the disease progressed (Fig. 3B). This property is particularly relevant to the use of MSCs as therapeutic delivery vehicles to target the wide spreading tumour nodules on the surface of the peritoneum.

In addition to tumour penetration, delivery of a high therapeutic payload is critical for achieving high potency in cancer killing. With only 2% therapeutic cells, CDUPRT-modified MSCs achieved significant killing efficiencies of 60, 70, and 80% for HT-29, Colo-205, and ES2 cells, respectively. When the therapeutic cell concentration was reduced further to 1% relative to cancer cells, CDUPRT-IFN modified MSCs demonstrated notably enhanced efficacy, resulting in killing efficiencies of 60% for HT-29, 70% for Colo-205, and a remarkable 95% for ES2.The superior anti-cancer effect of CDUPRT-IFN_MSC in the mice model further supports the notion that multi-transgene armed MSCs is a preferred therapeutic candidate for PC. Intriguingly, with virally engineered neural stem cells expressing CD and IFNb, Choi et al. demonstrated synergistic anti-cancer effect of such combination in some [51, 52] but not all [81–84] cancer cell lines. Using 100 µg/mL 5FC, only ~ 20% reduction in cell viability was observed at a 2:1 ratio of therapeutic cells to HT-29, and even increasing to a ratio of 6:1 did not improve cancer killing further [52]. A possible reason for this lack of cytoreductive efficiency is the limited payload of virally engineered cells [20].

The key to high payload is to maximise number of DNA copies in the cells. While transient transfection enables more than 10^4^ copies of DNA per cell [55], there have been several reports on the FDA’s position on restricting the integration of viral vectors into the host cell genome to fewer than 5 copies in cell and gene therapy productto mitigate the risk of oncogenesis [85]. This limitation on vector integration into the host genome results in a limited payload within the engineered cells. The high payload ensures the efficient conversion of 5FC to 5FU. We have previously shown that 100 µg/mL of 5FC and 20% CDUPRT engineered MSCs was as potent as 100 µg/mL 5FU in a coculture study with cancer cells [20]. In addition to the robust conversion of 5FC to 5FU, the expression level of secreted IFNb from our modified MSCs was found to be ~ 500 ng/mL. This represents the highest reported IFNb expression level, contrasting the typical pg/mL range in virally engineered MSC studies [86–88]. To our knowledge, this is the first report to demonstrate localized 5FU and IFNb treatment delivered by non-virally engineered MSCs.

It has not escaped our notice that this multi-armed MSCs could potentially exert further therapeutic effects through the induction of anti-cancer immunity. Studies are underway to demonstrate its potential effect in modulating the immune response in humanized mice models. Additionally, safety and efficacy studies will be conducted for investigational new drug application. In conclusion, we have shown that MSCs armed with a 5FU prodrug system and IFNb provided higher therapeutic efficacy than the 5FU prodrug system alone.

### Supplementary Information

Additional file 1: Design of experiment (DOE) for optimisation of non-viral transfection. Human MSCs were modified with increasing amounts of pDNA and polymer. The cells were harvested one day post-transfection and the (A) transfection efficiency and (B) cell viability was determined using flow cytometry. For viability measurements the cells were stained using 7-aminoactinomycin D (7AAD). A quadratic model was obtained using the DesignExpert (v.13) software (StatEase Inc., MN, USA) to obtain the optimized transfection parameters. (C) Images of cells transfection using the 16 conditions tested. Surface response graphs and images were obtained from a representative experiment run. Each DOE experiment was repeated at least thrice (n=3) to validate the results.

Additional file 2: Immunoblot analysis of CDUPRT and interferon-beta. MSCs were modified to express CDUPRT and IFNb. One day post transfection, conditioned media and cell lysate were analysed by immunoblotting. 20uL of conditioned media, or 20ug of protein from cell lysate was added to the respective wells. (A) and (B) were probed using anti-human IFNb and (C) using anti-eGFP antibody. Expected size. IFNb = 22.3kDa; CDUPRT-GFP = 68.1kDa; recombinant GFP = 26.2kDa. UT – Untransfected, L – Ladder, D1 – day 1 post-transfect, Ctrl – Control (for IFNb – recombinant human IFNb, Genscript #Z03109; for CDUPRT-GFP – Recombinant eGFP from e.coli). There was no detectable CDUPRT in the conditioned media (data not shown).

Additional file 3: Secretion of IFNb. Concentration (ng/mL) of IFNb secreted on day 1 post-transfection from native cells (Control), cells modified with CDUPRT only, and cells modified with CDUPRT-IFNb. Control and CDUPRT samples did not secrete IFNb and were below the detection limit of the assay.

Additional file 4: Interferon-β expressed in MSC is functional. (A) Schematic of the assay design for determining IFNb function. (B) MSC were transfected with CDUPRT, CDUPRT-IFNb or IFNb alone and allowed to express IFNb for two days. The supernatant was then collected and directly transferred into well plates containing A549 lung carcinoma cells. The conditioned medium was treated at a 1:1 ratio of conditioned medium to cell culture medium. One day later, the RNA was extracted and MxA, ADAR1 and ISG56 expression were detected using qPCR. All fold-changes were calculated using the ΔΔCT method using the untreated control and RPL19 as a biological normalizer. Here the untreated control refers to A549 cells without any treatment, untransfected control refers to the treatment with conditioned medium from native MSC and 10 ng/mL recombinant human IFNb (Genscript) was used as the positive control. All bars were represented as mean fold-change ±SD of three biological replicates (n=3).

Additional file 5: Karyotype of MSCs remain unchanged after modification. Karyotype of (A) non-modified MSCs, (B) CDUPRT modified MSCs, and (C) CDUPRT-IFNb modified MSCs. No abnormalities were observed.

Additional file 6: Conversion of 5FC to 5FU. Liquid chromatography tandem mass spectrometry (LC-MS/MS) was performed on supernatant samples collected from wells containing 0, 50, 750, and 7500 MSCs as indicated. The graph represents the average amount of 5FU detected from biological duplicates (n=2).

Additional file 7: 5FU sensitivity of various GBM cell lines. Six replicates of ES2 cell line (2500 cells) were plated. One day later, culture media were replaced with RPMI supplemented with 10% FBS and 5FU (0–100 μM). Cell viability was determined using Crystal Violet assay 48 hours later. The percentage of cell viability was calculated with no treatment control set at 100%.

Additional file 8: Differential activation of interferon stimulated genes (ISG) using modified MSCs. MSCs were transfected with CDUPRT, CDUPRT-IFNb. One day post transfection, 100 µg/mL 5FC was added to the transfected MSCs to allow for conversion of 5FC to 5FU for one day. The supernatant was then collected and directly transferred into well plates containing ES2 cells. The conditioned medium was treated at a 1:10 ratio of conditioned medium to cell culture medium. Three days later, the RNA was extracted and TNFSF10 (TRAIL), DR4, DR5, STAT1 and CXCL10 expressions were detected using qPCR. All fold-changes were calculated using the ΔΔCT method using the native control and RPL19 as a biological normalizer. Here, the native control refers to ES2 cells treated with conditioned media from native MSCs. All bars were represented as mean fold-change ± SD of three biological replicates (n=3). Significance was calculated using unpaired Students’ t-test. A p-value of less than 0.05 was considered significant. (*p<0.05, **p<0.01).

Additional file 9: Differential activation of immune cells in-vitro using conditioned medium from co-culture of CDUPRT-IFNb_MSC and colorectal cancer cells. CDUPRT-IFNb_MSC and CDUPRT_MSC were co-cultured with (A) COLO 205 or (B) HT-29 colorectal adenocarcinoma cell lines at a ratio of 1 MSC to 5 or 10 cancer cells. One day later, co-cultures were treated with or without 150 µg/mL 5FC. Percentage killing was assessed 5 days post-treatment with 5FC by harvesting and counting of viable cells. Cells co-cultured with CDUPRT_MSC at different ratios without 5FC treatment was used as the respective controls to calculate cancer killing percentage using the formula: Percentage Killing = 1-(sample/control) * 100%. Separately, the co-culture supernatant was collected from COLO 205 at 24 hours and 48 hours after the addition of 5FC for a THP-1 stimulation assay. THP-1 cells were plated onto 96well plates and treated with respective supernatant samples as indicated. After overnight treatment, the cells were harvested and stained for (C) CD80, (D) CD40, (E) CD86, and (F) HLA-DR. All graphs were represented as mean ±SD from at least three biological replicates (n=3). Significance was calculated using two-way ANOVA with Tukey’s correction. A p-value of less than 0.05 was considered significant. (*p<0.05, **p<0.01, ***p<0.001, ****p<0.00001).

Additional file 10.

## Data Availability

The authors confirm that the data supporting the findings of this study are available within the article and its supplementary materials.

## Abbreviations

MSCs: Mesenchymal stem cells
CDUPRT: Cytosine deaminase and uracil phosphoribosyl transferase
GFP: Green fluorescent protein
IFNb: Interferon-beta
PC: Peritoneal carcinomatosis
5FU: 5-Fluorouracil
5FC: 5-Flucytosine
OS: Overall survival
CRS: Cytoreductive surgery
HIPEC: Hyperthermic intraperitoneal chemotherapy
HLA-DR: Human Leukocyte Antigen-DR
TMRE: Tetramethylrhodamine ethyl ester perchlorate
