# Supplementary figures and images for: Enhanced anti-tumor efficacy with multi-transgene armed mesenchymal stem cells for treating peritoneal carcinomatosis
Source: J Transl Med. 2024 May 15;22:463. doi: 10.1186/s12967-024-05278-5 (PMC11097589; doi:10.1186/s12967-024-05278-5)

## Slide 1
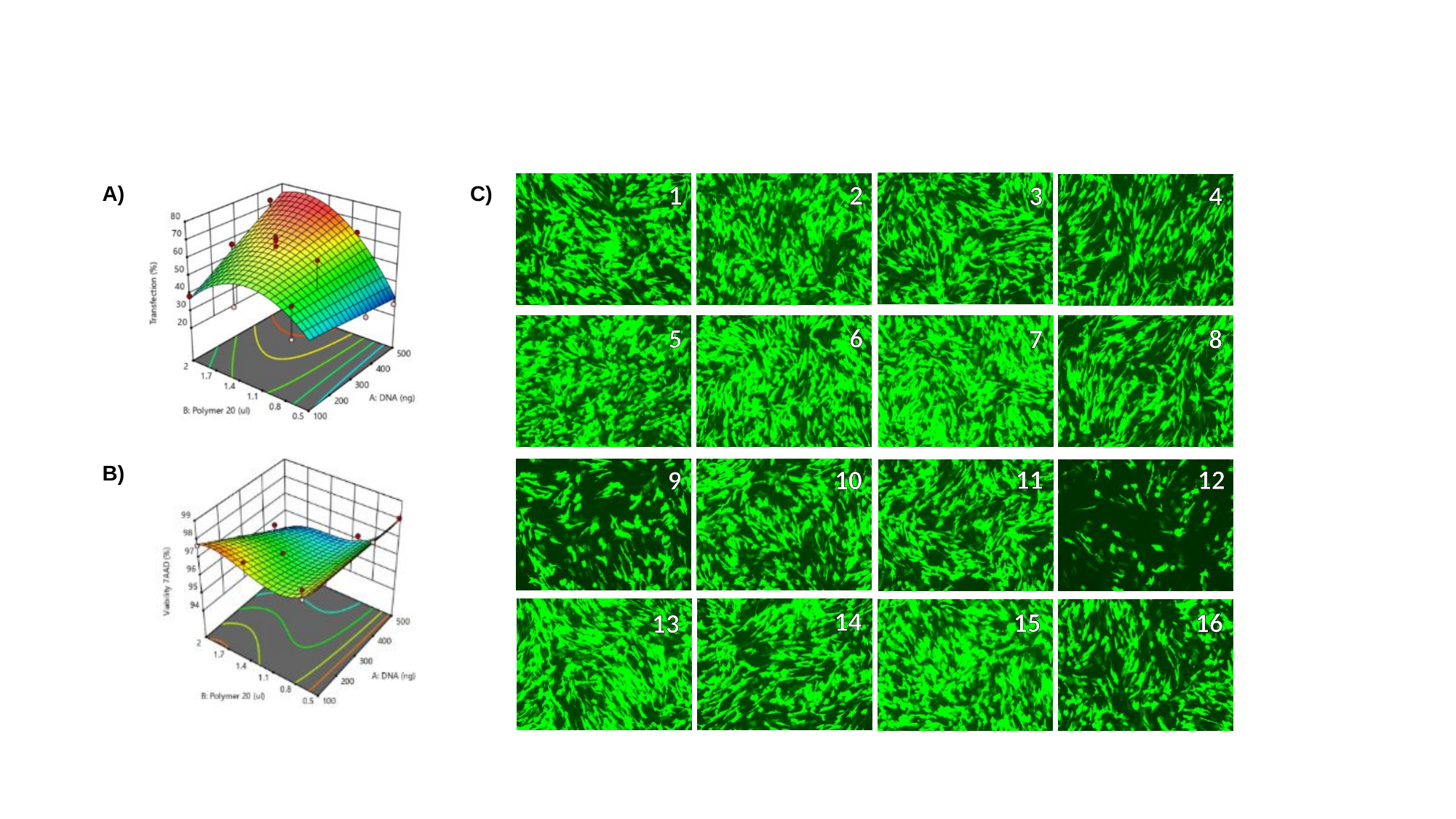

2
1
3
4
A)
C)
6
5
7
8
B)
11
12
10
9
14
15
16
13

Supplement: Supplementary file 1 — Additional file 1: Design of experiment (DOE) for optimisation of non-viral transfection. Human MSCs were modified with increasing amounts of pDNA and polymer. The cells were harvested one day post-transfection and the (A) transfection efficiency and (B) cell viability was determined using flow cytometry. For viability measurements the cells were stained using 7-aminoactinomycin D (7AAD). A quadratic model was obtained using the DesignExpert (v.13) software (StatEase Inc., MN, USA) to obtain the optimized transfection parameters. (C) Images of cells transfection using the 16 conditions tested. Surface response graphs and images were obtained from a representative experiment run. Each DOE experiment was repeated at least thrice (n=3) to validate the results. [file 12967_2024_5278_MOESM1_ESM.pptx]

## Slide 1
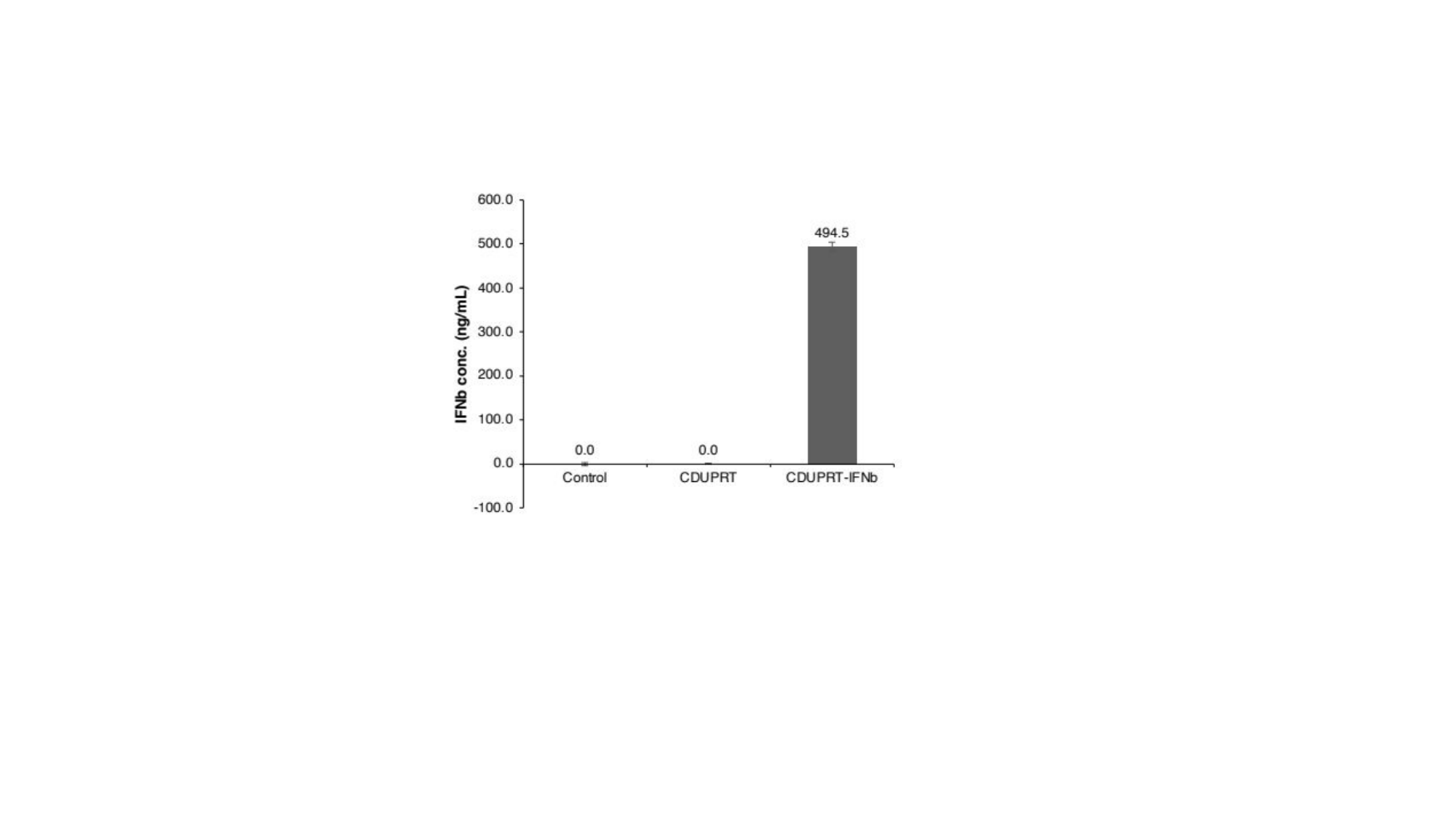

Supplement: Supplementary file 3 — Additional file 3: Secretion of IFNb. Concentration (ng/mL) of IFNb secreted on day 1 post-transfection from native cells (Control), cells modified with CDUPRT only, and cells modified with CDUPRT-IFNb. Control and CDUPRT samples did not secrete IFNb and were below the detection limit of the assay. [file 12967_2024_5278_MOESM3_ESM.pptx]

## Slide 1
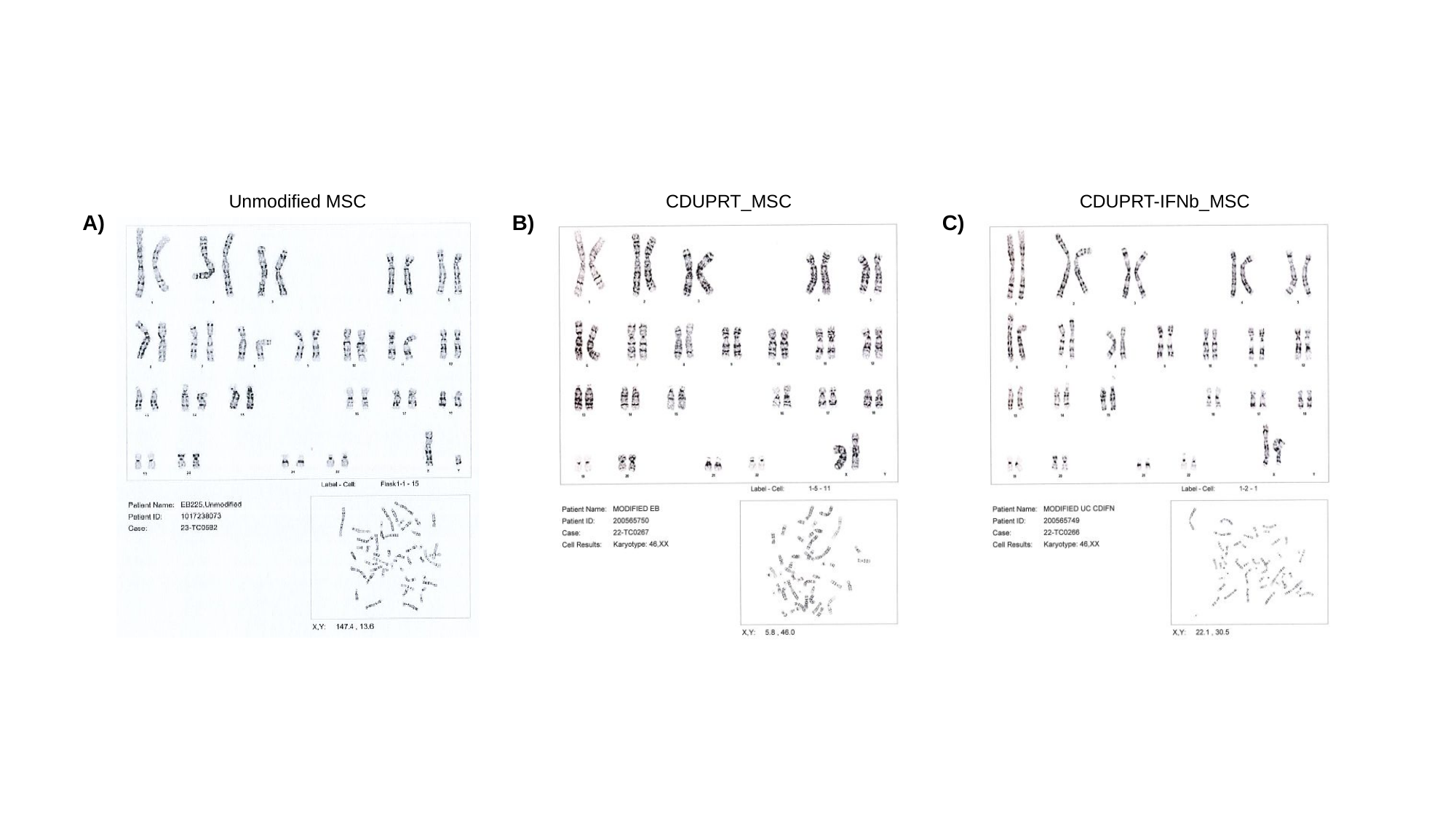

Unmodified MSC
CDUPRT_MSC
CDUPRT-IFNb_MSC
A)
B)
C)

Supplement: Supplementary file 5 — Additional file 5: Karyotype of MSCs remain unchanged after modification. Karyotype of (A) non-modified MSCs, (B) CDUPRT modified MSCs, and (C) CDUPRT-IFNb modified MSCs. No abnormalities were observed. [file 12967_2024_5278_MOESM5_ESM.pptx]

## Slide 1
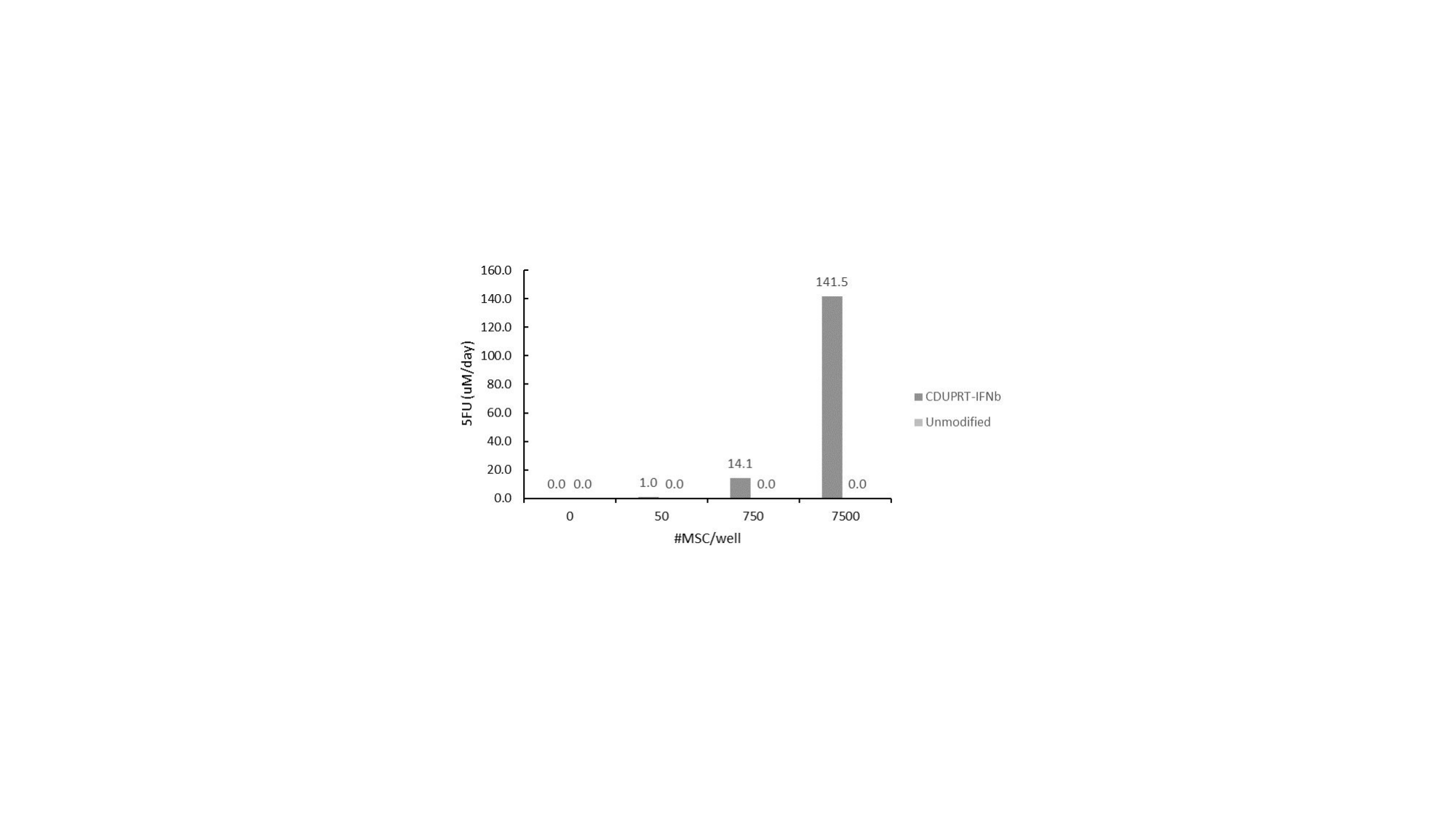

Supplement: Supplementary file 6 — Additional file 6: Conversion of 5FC to 5FU. Liquid chromatography tandem mass spectrometry (LC-MS/MS) was performed on supernatant samples collected from wells containing 0, 50, 750, and 7500 MSCs as indicated. The graph represents the average amount of 5FU detected from biological duplicates (n=2). [file 12967_2024_5278_MOESM6_ESM.pptx]

## Slide 1
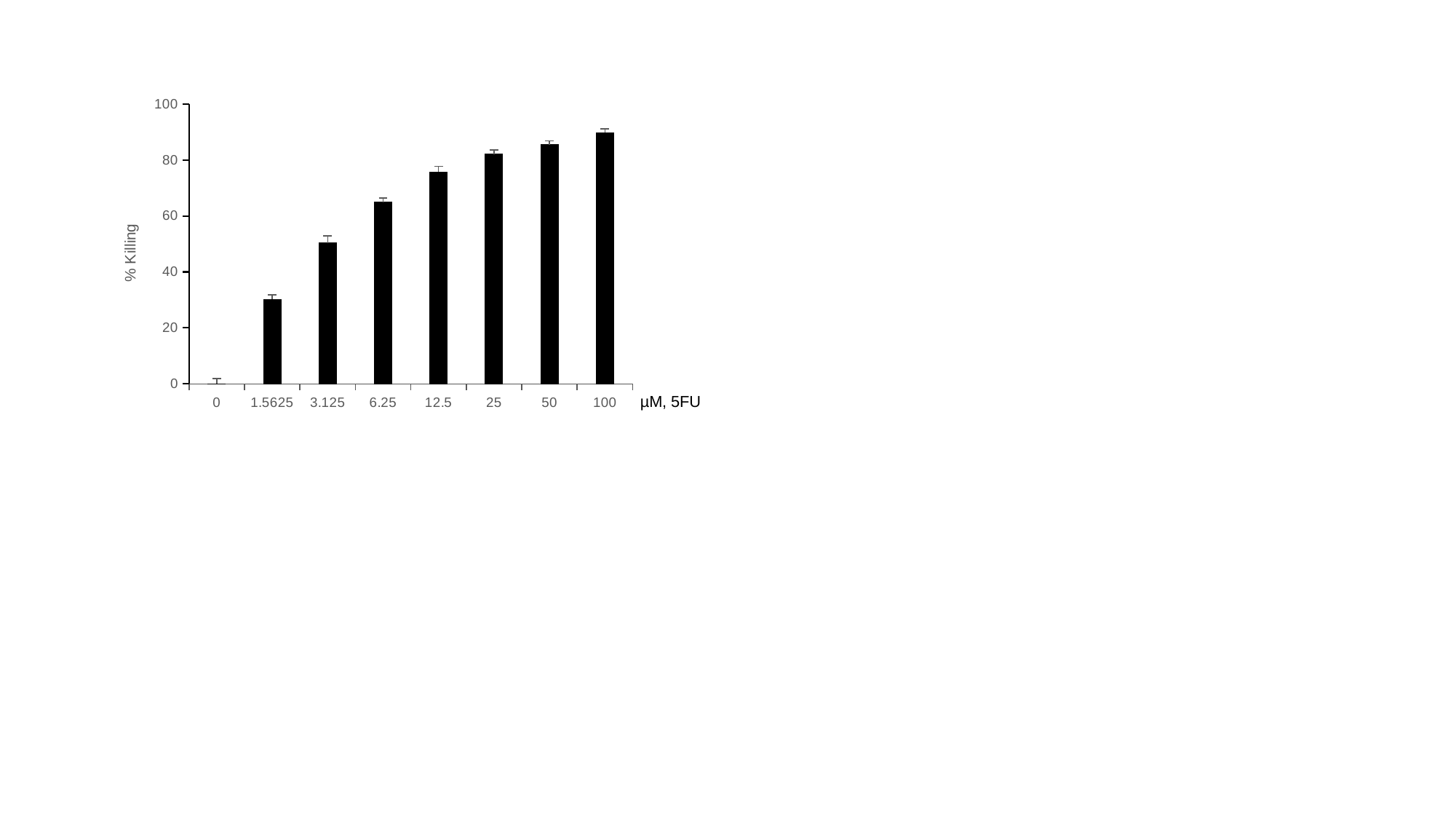

### Chart
| Category | kill |
|---|---|
| 0 | 0.0 |
| 1.5625 | 30.22936409833814 |
| 3.125 | 50.41889850295289 |
| 6.25 | 64.97733827770911 |
| 12.5 | 75.70388682873231 |
| 25 | 82.07663782447464 |
| 50 | 85.52396648811975 |
| 100 | 89.72668589479468 |µM, 5FU

Supplement: Supplementary file 7 — Additional file 7: 5FU sensitivity of various GBM cell lines. Six replicates of ES2 cell line (2500 cells) were plated. One day later, culture media were replaced with RPMI supplemented with 10% FBS and 5FU (0–100 μM). Cell viability was determined using Crystal Violet assay 48 hours later. The percentage of cell viability was calculated with no treatment control set at 100%. [file 12967_2024_5278_MOESM7_ESM.pptx]

## Slide 1
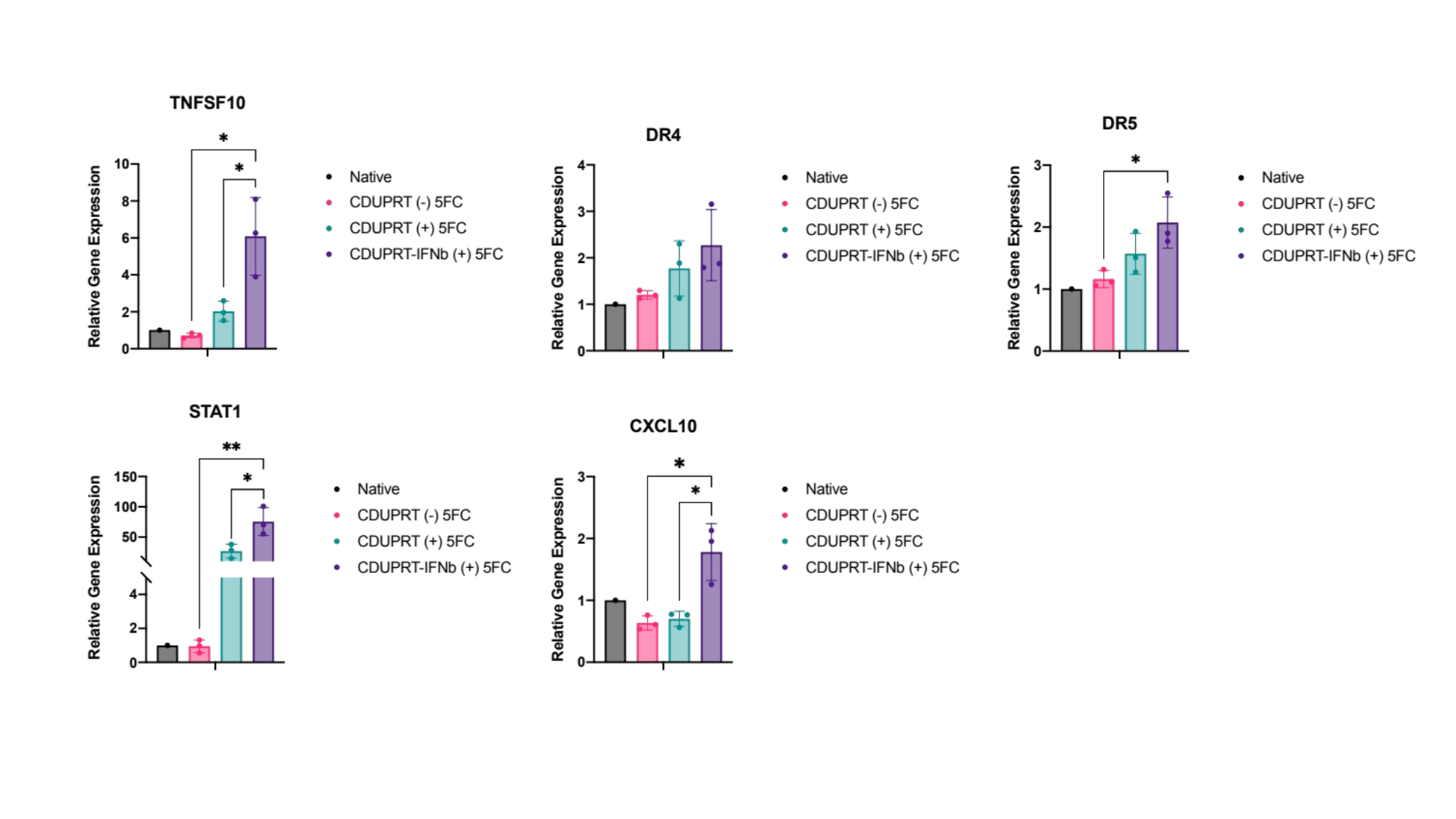

Supplement: Supplementary file 8 — Additional file 8: Differential activation of interferon stimulated genes (ISG) using modified MSCs. MSCs were transfected with CDUPRT, CDUPRT-IFNb. One day post transfection, 100 µg/mL 5FC was added to the transfected MSCs to allow for conversion of 5FC to 5FU for one day. The supernatant was then collected and directly transferred into well plates containing ES2 cells. The conditioned medium was treated at a 1:10 ratio of conditioned medium to cell culture medium. Three days later, the RNA was extracted and TNFSF10 (TRAIL), DR4, DR5, STAT1 and CXCL10 expressions were detected using qPCR. All fold-changes were calculated using the ΔΔCT method using the native control and RPL19 as a biological normalizer. Here, the native control refers to ES2 cells treated with conditioned media from native MSCs. All bars were represented as mean fold-change ± SD of three biological replicates (n=3). Significance was calculated using unpaired Students’ t-test. A p-value of less than 0.05 was considered significant. (*p<0.05, **p<0.01). [file 12967_2024_5278_MOESM8_ESM.pptx]

## Slide 1
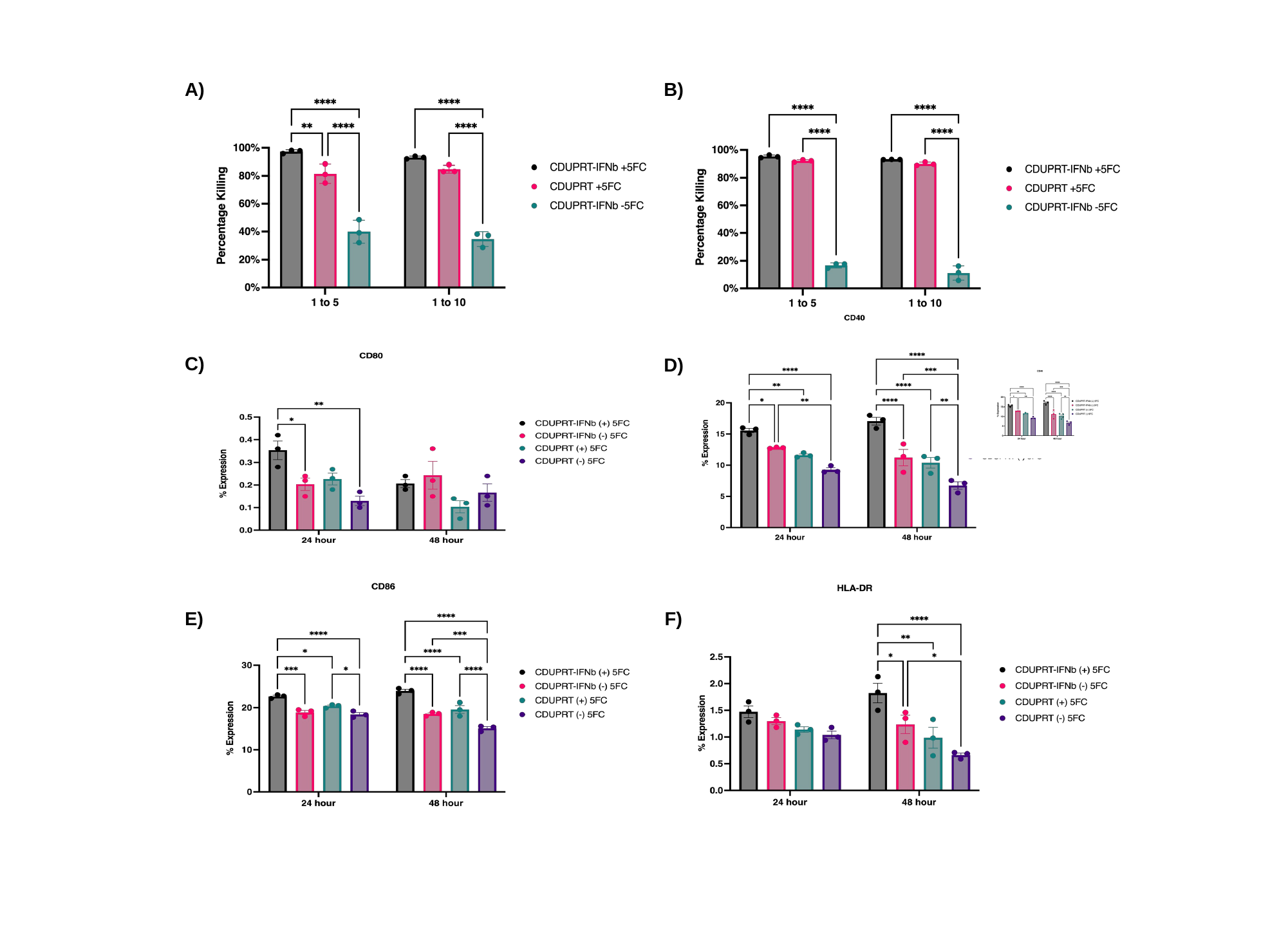

A)
B)
C)
D)
E)
F)

Supplement: Supplementary file 9 — Additional file 9: Differential activation of immune cells in-vitro using conditioned medium from co-culture of CDUPRT-IFNb_MSC and colorectal cancer cells. CDUPRT-IFNb_MSC and CDUPRT_MSC were co-cultured with (A) COLO 205 or (B) HT-29 colorectal adenocarcinoma cell lines at a ratio of 1 MSC to 5 or 10 cancer cells. One day later, co-cultures were treated with or without 150 µg/mL 5FC. Percentage killing was assessed 5 days post-treatment with 5FC by harvesting and counting of viable cells. Cells co-cultured with CDUPRT_MSC at different ratios without 5FC treatment was used as the respective controls to calculate cancer killing percentage using the formula: Percentage Killing = 1-(sample/control) * 100%. Separately, the co-culture supernatant was collected from COLO 205 at 24 hours and 48 hours after the addition of 5FC for a THP-1 stimulation assay. THP-1 cells were plated onto 96well plates and treated with respective supernatant samples as indicated. After overnight treatment, the cells were harvested and stained for (C) CD80, (D) CD40, (E) CD86, and (F) HLA-DR. All graphs were represented as mean ±SD from at least three biological replicates (n=3). Significance was calculated using two-way ANOVA with Tukey’s correction. A p-value of less than 0.05 was considered significant. (*p<0.05, **p<0.01, ***p<0.001, ****p<0.00001). [file 12967_2024_5278_MOESM9_ESM.pptx]
