# Supplementary material for: Enhanced anti-tumor efficacy with multi-transgene armed mesenchymal stem cells for treating peritoneal carcinomatosis
Source: J Transl Med. 2024 May 15;22:463. doi: 10.1186/s12967-024-05278-5 (PMC11097589; doi:10.1186/s12967-024-05278-5)
Supplement: Supplementary file 2 — Additional file 2: Immunoblot analysis of CDUPRT and interferon-beta. MSCs were modified to express CDUPRT and IFNb. One day post transfection, conditioned media and cell lysate were analysed by immunoblotting. 20uL of conditioned media, or 20ug of protein from cell lysate was added to the respective wells. (A) and (B) were probed using anti-human IFNb and (C) using anti-eGFP antibody. Expected size. IFNb = 22.3kDa; CDUPRT-GFP = 68.1kDa; recombinant GFP = 26.2kDa. UT – Untransfected, L – Ladder, D1 – day 1 post-transfect, Ctrl – Control (for IFNb – recombinant human IFNb, Genscript #Z03109; for CDUPRT-GFP – Recombinant eGFP from e.coli). There was no detectable CDUPRT in the conditioned media (data not shown). [file 12967_2024_5278_MOESM2_ESM.pptx]

## Slide 1
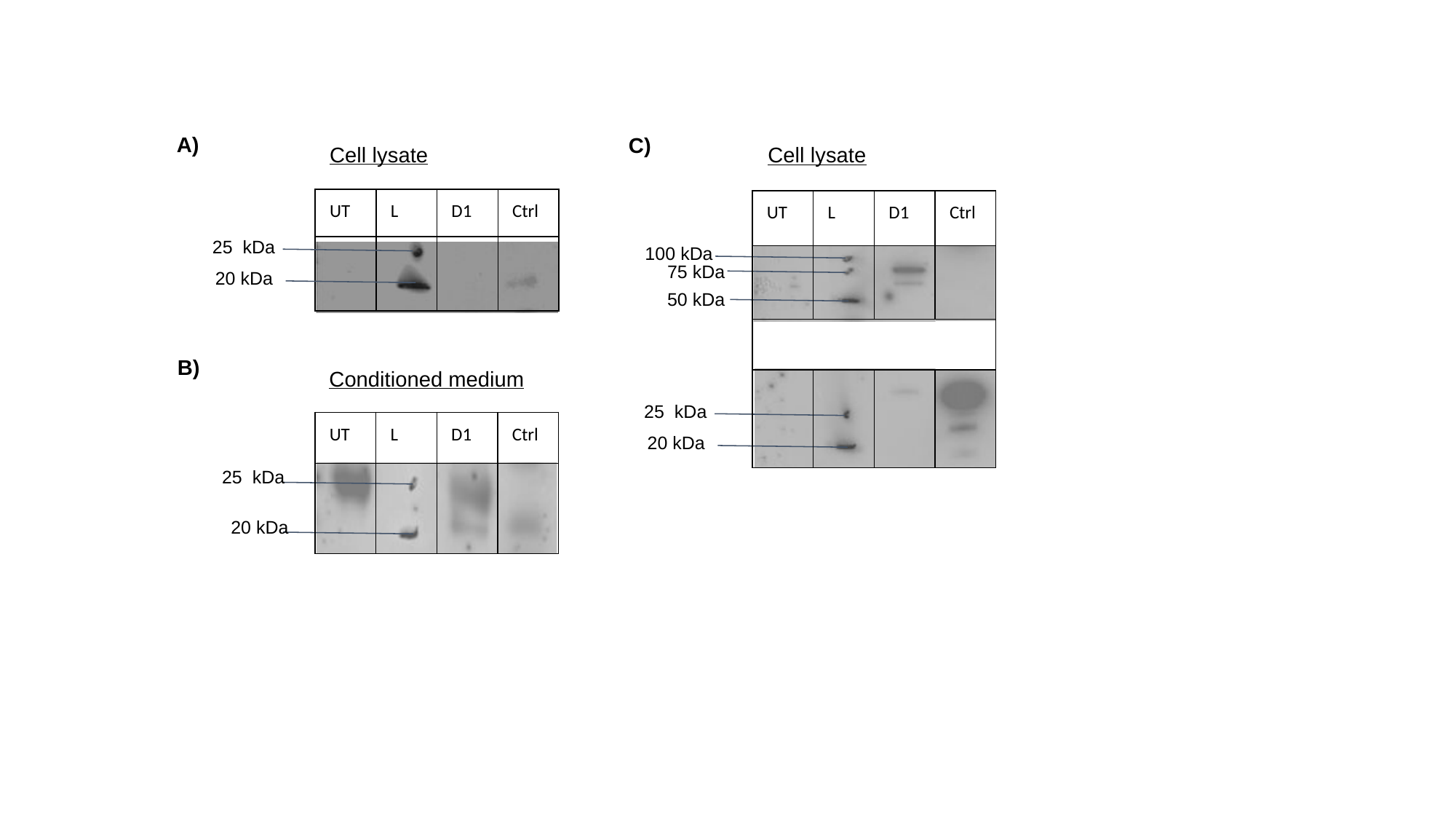

A)
C)
Cell lysate
Cell lysate
| UT | L | D1 | Ctrl |
| --- | --- | --- | --- |
| | | | |
| UT | L | D1 | Ctrl |
| --- | --- | --- | --- |
| | | | |
| | | | |
| | | | |
25 kDa
100 kDa
75 kDa
20 kDa
50 kDa
B)
Conditioned medium
25 kDa
| UT | L | D1 | Ctrl |
| --- | --- | --- | --- |
| | | | |
20 kDa
25 kDa
20 kDa
