# Supplementary material for: Enhanced anti-tumor efficacy with multi-transgene armed mesenchymal stem cells for treating peritoneal carcinomatosis
Source: J Transl Med. 2024 May 15;22:463. doi: 10.1186/s12967-024-05278-5 (PMC11097589; doi:10.1186/s12967-024-05278-5)
Supplement: Supplementary file 4 — Additional file 4: Interferon-β expressed in MSC is functional. (A) Schematic of the assay design for determining IFNb function. (B) MSC were transfected with CDUPRT, CDUPRT-IFNb or IFNb alone and allowed to express IFNb for two days. The supernatant was then collected and directly transferred into well plates containing A549 lung carcinoma cells. The conditioned medium was treated at a 1:1 ratio of conditioned medium to cell culture medium. One day later, the RNA was extracted and MxA, ADAR1 and ISG56 expression were detected using qPCR. All fold-changes were calculated using the ΔΔCT method using the untreated control and RPL19 as a biological normalizer. Here the untreated control refers to A549 cells without any treatment, untransfected control refers to the treatment with conditioned medium from native MSC and 10 ng/mL recombinant human IFNb (Genscript) was used as the positive control. All bars were represented as mean fold-change ±SD of three biological replicates (n=3). [file 12967_2024_5278_MOESM4_ESM.pptx]

## Slide 1
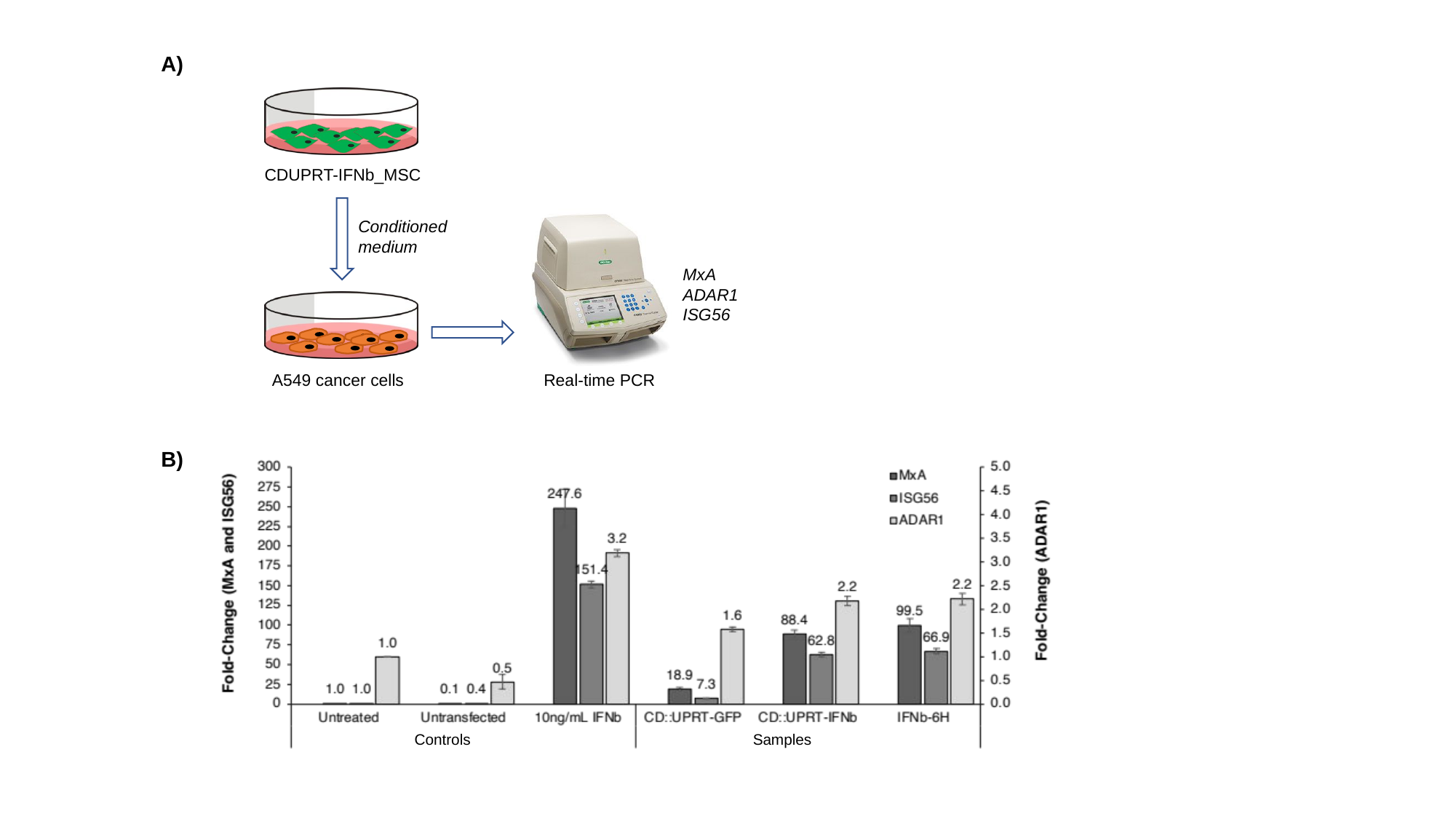

A)
CDUPRT-IFNb_MSC
Conditioned medium
MxA
ADAR1
ISG56
A549 cancer cells
Real-time PCR
Samples
Controls
B)
