# Supplementary material for: Enhanced anti-tumor efficacy with multi-transgene armed mesenchymal stem cells for treating peritoneal carcinomatosis
Source: J Transl Med. 2024 May 15;22:463. doi: 10.1186/s12967-024-05278-5 (PMC11097589; doi:10.1186/s12967-024-05278-5)
Supplement: Supplementary file 10 — Additional file 10. [file 12967_2024_5278_MOESM10_ESM.docx]

**Supplementary materials and methods**

**Design of experiment (DOE)**

The design of experiment (DOE) was carried out in 48-well plate format. A quadratic model was obtained using the DesignExpert (v.13) software (StatEase Inc., MN, USA) to obtain the optimized transfection parameters. Briefly, plasmid complex at a total volume of 20 μL/cm², consist of 16 conditions of different amounts of pDNA and Polyethylenimine MAX (Polyscience; 1 mg/mL) and DPBS, was incubated at room temperature for 15 min. The plasmid complex was then added to MSCs (150000 cells/cm²) supplemented with 500 ng/mL Lipofectamine™ 2000 Transfection Reagent (ThermoFisher Scientific) and 0.5 μM Vorinostat (Histone deacetylase inhibitor; HDACi, BioVision) in complete medium. The cells were harvested one day post-transfection. Cell images were taken with EVOS M7000 Imaging System (Thermo Fisher Scientific) equipped with GFP (Ex470/Em510) fluorescent light cube. For flow cytometric analyses, cells were washed by DPBS, trypsinized using TrypLE Express. Cells were then stained with 5µL/mL of 7-aminoactinomycin D (7AAD, Tonbo Biosciences) for 10 min at room temperature. Percentage of fluorescent positive cells and cell viability were quantified by Attune NxT Flow Cytometer system (ThermoFisher Scientific), and the raw data was analysed using non-modified MSCs as negative controls at <0.8%, using Invitrogen Attune NxT software (ThermoFisher Scientific).

**Genetic stability study (karyotyping)**

Unmodified (Native) MSCs, MSCs modified with CDUPRT, and MSCs modified with CDUPRT-IFNb were prepared in T25 flasks and analysed by a qualified pathologist from the National University Hospital (NUH) Singapore, Department of Laboratory Medicine, Cytogenetics Laboratory.

**Western blot**

To analyze IFNb protein expression, cell culture supernatant was collected, and cells were harvested and lysed with lysis buffer (150 mM sodium chloride, 1.0% NP-40, 50 mM Tris pH 8.0) supplemented with protease inhibitor cocktail (Roche). The protein samples of supernatant and whole cell lysate were loaded onto 8% polyacrylamide gels and analyzed by means of immunoblotting with rabbit anti-human IFNb (PeproTech, #500-P32B) and mouse anti-eGFP (Abcam, #Ab184601), respectively.

**ELISA**

Transfection was carried out in 6-well plate format. Cell culture supernatant was collected on day 1 post-transfection and stored at -80°C till needed for use. ELISA was performed using a commercial kit, according to the manufacturer's protocol (Human IFN-Beta ELISA Kit, PBL Assay Science).

**qPCR**

The transcript level of IFN-stimulated genes was analyzed by qPCR using the Real Time PCR System (Bio-Rad). MSC were transfected with CDUPRT, CDUPRT-IFNb or IFNb alone and allowed to express the transgene for one to two days. The supernatant was then collected and directly transferred into well plates containing A549 or ES-2 cell lines. The conditioned medium was treated at a 1:1 and 1:10 ratio of conditioned medium to cell culture medium for A549 and ES2 cells, respectively. One to three days later, the RNA was extracted and the expression of the genes of interest were detected using qPCR.

**In-vitro estimation of 5FC to 5FU conversion**

Unmodified MSCs or MSCs modified with CDUPRT-IFNb were plated at 0, 50, 750, and 7500 cells per well in a 96-well plate. The cells were allowed to adhere overnight before treatment with 100 µg/mL 5FC. One days post treatment, the supernatant was collected and spun down to remove any cellular debris. An Agilent Triple Quadrupole LCMS-6495C with AJS ESI ion source was applied to analyze 5-FU within the cell culture supernatant. A ZORBAX Eclipse Plus C18 column (2.1 × 100 mm, 1.8 Micron, Agilent) was used to separate 5-FU from other components. The oven temperature was 40 °C. MilliQ water (0.1% formic acid) and acetonitrile (0.1% formic acid) were used as mobile phase A and mobile phase B, respectively. The total run time was 14 min. Multiple reaction monitoring (MRM) mode was applied to analyze 5-FU (129.0 -> 42.1, negative mode).

**THP-1 stimulation assay**

500 cells/well of COLO-205-LUC-GFP, and 2000 cells/well of HT29 were seeded in 96-well plates. Four hours later, CDUPRT_MSCs or CDUPRT-IFNb_MSCs were plated to the cancer cell culture at the MSC to cancer ratios of 1 MSC to 5 or 10 cancer cells. One day later, co-cultures were treated with or without 150 µg/mL 5FC. The co-culture supernatant was collected from COLO-205 at 24 hours and 48 hours after the addition of 5FC for THP-1 stimulation assay. THP-1 cells were plated onto 96well plates and treated with respective supernatant samples as indicated. After overnight treatment, the cells were harvested and stained for CD80, CD40, CD86, and HLA-DR.
